# Sex hormones in the nucleus incertus: a novel perspective on sex/gender differences in memory and mood dysfunction in Alzheimer’s disease

**DOI:** 10.3389/fnagi.2026.1852733

**Published:** 2026-09-03

**Authors:** Camila de Ávila, Ayanabha Chakraborti, Andrew L. Gundlach, Lisa Y. Maeng

**Affiliations:** 1 Department of Translational Neurosciences, University of Arizona College of Medicine – Phoenix, Phoenix, AZ, United States; 2 The Florey, The University of Melbourne, Parkville, VIC, Australia; 3 Florey Department of Neuroscience and Mental Health, The University of Melbourne, Parkville, VIC, Australia; 4 Department of Anatomy and Physiology, The University of Melbourne, Parkville, VIC, Australia; 5 Developmental and Brain Sciences Program, Department of Psychology, University of Massachusetts Boston, Boston, MA, United States

**Keywords:** estrogen, learning and memory, mild cognitive impairment, relaxin-3, RXFP3, steroid hormones, stress

## Abstract

About 60% of the individuals living with Alzheimer’s disease (AD) in the United States are women. Increasing evidence points to a role for sex hormones, especially estrogen, in making women more vulnerable to AD than men. Several brainstem signaling pathways, as well as multiple neuropeptide modulators and their receptors, are implicated in AD pathology and therapeutic possibilities. However, there is limited research evaluating how the modulatory effects of sex hormones on mood and cognition interact with these brainstem systems. This review seeks to examine possible connections between the nucleus incertus (NI) relaxin-3/relaxin-family peptide receptor 3 (RLN3/RXFP3) system and sex hormone influences in AD to encourage further investigation into how sex hormones may modulate this pathway and contribute to sex differences in disease risk and progression. Understanding how hormone-based interventions, such as hormone replacement therapy (HRT) and hormonal contraceptives (HC), affect NI RLN3/RXFP3 signaling may provide novel insights into how these treatments can slow AD-associated cognitive decline. It is hoped that this data and concept synthesis will drive detailed investigations of these circuits and support the development of sex/gender-informed therapeutic strategies that better reflect the heterogeneity of AD dementia.

## Graphical Abstract

**Figure:**
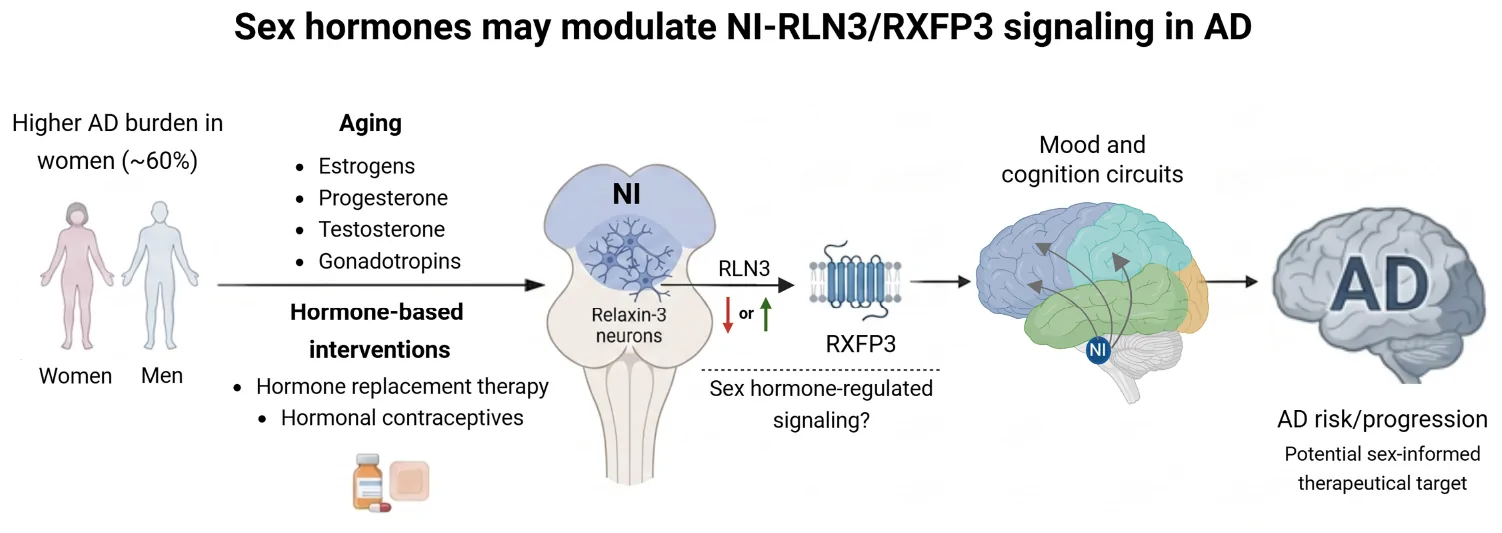
Sex hormones may modulate NI relaxin-3/RXFP3 signaling in Alzheimer’s disease (AD). Created in BioRender.com.

## 1 Alzheimer’s disease: global burden and knowledge gaps

### 1.1 Neuropathological hallmarks

Alzheimer’s disease (AD) is the most common cause of dementia worldwide and imposes a substantial burden on global public health systems (Knopman et al., 2021; Xiaopeng et al., 2025). Clinically, AD causes gradual cognitive decline, and many individuals also experience neuropsychiatric symptoms such as anxiety and depression that further increase disease-related disability (Zhang J. et al., 2024; Zhang N. K. et al., 2024). In the United States, ∼7 million adults aged 65 and older have AD-related dementia, and projections indicate this may reach ∼14 million by 2060 (Alzheimers Dementia, 2024). At the neuropathological level, AD is characterized by the accumulation of amyloid-beta (Aβ) plaques and neurofibrillary tangles composed of hyperphosphorylated tau, both of which can disrupt neuronal communication and networks that support memory and other cognitive functions (Busche and Hyman, 2020; Spires-Jones and Hyman, 2014; Trejo-Lopez et al., 2022). Current medications offer limited benefit and generally do not influence the biological progression of the disease (Sharma et al., 2025). Therefore, a better understanding of the temporal sequence of cellular and molecular events initiating and driving AD pathology and the precise cell types involved is needed to guide the development of more effective interventions.

Accumulating evidence from both animal and human studies indicates that AD affects males/men and females/women differently (Lopez-Lee et al., 2024; Pike, 2017). Women account for nearly two-thirds of diagnosed cases and often display faster cognitive decline and a heavier pathological load than men (Lopez-Lee et al., 2024). Genetic influences contribute to this difference; for instance, the apolipoprotein E (APOE) ε4 allele typically has stronger effects in females (Altmann et al., 2014). Hormonal status, sex-related differences in brain organization, and broader sociocultural factors may also contribute (Kolahchi et al., 2024). Although several factors have been identified, the precise biological pathways underlying these sex-linked patterns in AD are not fully characterized (Guo et al., 2023), and advancing our knowledge of the neurobiology involved may assist in developing approaches that account for sex-specific aspects of the disease.

### 1.2 Role of the brainstem-limbic system

Most research on AD pathology and symptoms has long centered on forebrain regions, particularly the hippocampus and cortex (Abubakar et al., 2022; Rao et al., 2022). However, other studies have highlighted that various brainstem nuclei and neural networks, involved in autonomic and complex functions, are affected during the early stages of AD (Braak et al., 2011). For example, the locus coeruleus and the raphe nuclei regulate attention/arousal and mood/emotion, respectively (Hornung, 2003; Sara and Bouret, 2012). These nuclei can display elevated hallmark AD-related pathology (Aβ and tau), in Braak stages I–II (Braak et al., 2011; Busche and Hyman, 2020). In addition, the brainstem contains the nucleus incertus (NI), which also has a role in controlling arousal, stress responses, emotion, and memory via its direct/indirect modulation of key forebrain circuits in the septohippocampal system, amygdala, and cortex in vertebrates (see further details below).

Notably, the NI contains mainly long-projection GABAergic neurons (Goto et al., 2001; Olucha-Bordonau et al., 2003) that express several neuropeptides, including relaxin-3 (RLN3), neuromedin-B, and cholecystokinin (Lu et al., 2020; Ma et al., 2007; Miyamoto et al., 2008; Nardone et al., 2024; Nasirova et al., 2020; Olucha-Bordonau et al., 2003; Smith et al., 2010; Szlaga et al., 2022). Neurons in this area send projections to several forebrain targets, including the hippocampus, hypothalamus, prefrontal cortex, and amygdala (García-Díaz et al., 2021; Gil-Miravet et al., 2023; Goto et al., 2001; Ma et al., 2007; Olucha-Bordonau et al., 2003; Szõnyi et al., 2019), and rodent studies have linked this pathway to the regulation of arousal, stress responses, feeding and metabolism, sleep-wake patterns, and forms of hippocampal theta activity relevant for learning and memory (Gil-Miravet et al., 2021; Lu et al., 2020; Ma et al., 2009, 2013, 2017a; 2017b; Olucha-Bordonau et al., 2018; Ryan et al., 2011; Smith et al., 2011; Szõnyi et al., 2019). For example, in rodent models, exposure to stress activates NI neurons (Banerjee et al., 2010; Calvez et al., 2016b,c; Gugula et al., 2025; Lenglos et al., 2013; Rajkumar et al., 2016), and elevates RLN3 production (Banerjee et al., 2010; Ma et al., 2013); and RLN3 signaling via the G_*i*/*o*_-protein-coupled receptor, relaxin-family peptide receptor 3 (RXFP3), can alter neural networks involved in emotional and cognitive functions (Ma et al., 2009, 2017b; Olucha-Bordonau et al., 2018; Ryan et al., 2013), which are also disturbed in early AD (Abubakar et al., 2022; Kumar et al., 2017; Lucey et al., 2021; Rao et al., 2022; Zhang N. K. et al., 2024). Critically, characteristic RLN3-expressing neurons have recently been identified in the human NI (de Ávila et al., 2024), supporting the possibility that NI-related systems modulate human memory and emotion and may be relevant to aging, dementia, and AD.

Together with other major circuits, this brainstem-limbic system is sensitive to gonadal steroids (Littlejohn et al., 2020; McEwen et al., 2012), and studies have reported sex-related variations in RLN3 expression and sex-dependent behavioral and endocrine responses of this system to chronic stress and dietary conditions (Calvez et al., 2016c; de Ávila et al., 2021; Gugula et al., 2025; Lenglos et al., 2013, 2015). Thus, as the NI integrates stress and memory-related information, and these functions differ between males and females, changes in NI RLN3/RXFP3 signaling may influence how these circuits respond to early AD-related changes. These observations suggest sex-dependent alterations within such brainstem neuromodulatory pathways could contribute to distinct patterns of AD disease progression, emphasizing the importance of studies within a sex-based framework.

## 2 Sex/gender differences

### 2.1 Clinical presentation

About 60% of the estimated seven million people living with AD in the USA today are women (Alzheimers Dementia, 2024; Rajan et al., 2021). The overall lifetime risk for AD is also higher in women (1-in-5) than in men (1-in-10) (Alzheimers Dementia, 2024; Chêne et al., 2015). Given that AD impacts older individuals, the bias toward women may be partly due to their increased longevity. However, increasing evidence suggests other factors may be driving this disparity in prevalence, and it remains unclear why women are twice as likely as men to develop AD (Moutinho, 2025). Sex and gender differences significantly influence AD symptoms, diagnosis, progression, and treatment outcomes.^1^ Women display stronger verbal memory than men in early-stage AD (Sundermann et al., 2016), often leading to late diagnosis and fewer opportunities for early intervention. However, while women can maintain normal cognitive performance in early-stage AD, once they reach the mild cognitive impairment (MCI) stage, they display more rapid cognitive decline to the dementia stage (Emrani and Sundermann, 2025). In addition, female patients tend to exhibit neuropsychiatric disorders such as depression and anxiety, both before and during AD progression (Eikelboom et al., 2022), posing additional challenges to optimal diagnosis and treatment.

### 2.2 Neuropathology

Sex/gender differences are well-established in both the clinical symptoms of AD and the brain and biomarker changes occurring over the disease continuum (Barnes et al., 2005). AD is characterized by neuropathological changes including the accumulation of cortical Aβ plaques and tau tangles throughout the cortex (Frisoni et al., 2025). Women carry a higher burden and faster accumulation of neurofibrillary tau tangles, with stronger associations between Aβ and tau compared to men (Boccalini et al., 2025; Buckley et al., 2019; Smith et al., 2020). In addition, female AD patients display profound changes in brain structural integrity associated with AD-related cognitive decline, whereby women with faster cognitive deterioration have significant cortical thinning (Boccalini et al., 2025), smaller hippocampi (Apostolova et al., 2006; Lin et al., 2015), global brain atrophy (Hua et al., 2010; Skup et al., 2011), and stronger associations with decreased brain volume in women with MCI that progressed to AD compared to those that did not (Fernández et al., 2024). These more pronounced effects of AD on the female brain may be partly driven by the APOE ε4 allele, which has been shown to disrupt brain structure and metabolism more strongly in women than in men (Sampedro et al., 2015). In addition to structural brain differences, men and women exhibit functional differences during AD progression. Alterations in low-frequency activity, such as delta and theta rhythms, and decreased high-frequency alpha and beta activity, are reported as critical features of increased risk for AD progression (Fernández et al., 2024; Jeong, 2004). Fernández et al. (2024) reported that compared to females with MCI who did not progress to AD, MCI females who progressed to AD displayed increased low-frequency (theta) power, which was associated with accelerated AD progression and more pronounced AD-related cognitive and brain changes. These patterns suggest that sex/gender is a significant, yet often underappreciated, determinant of AD risk, trajectory, and symptomatology (Aggarwal and Mielke, 2023).

### 2.3 Role of steroid hormones in AD pathogenesis

Although multiple factors, including lifestyle, genetics, and sociocultural influences, likely contribute to sex/gender differences in AD, both preclinical and clinical evidence indicate that steroid hormones play a key role [see Lopez-Lee et al. (2024) for review]. Steroid hormones, such as estrogen in its prominent form, estradiol (E2), progesterone, and testosterone display neuroprotective properties (Siddiqui et al., 2016), and affect cognition by modulating brain regions critical for mood and memory, including the hippocampus, amygdala, and prefrontal cortex [for review, see (Bencker et al., 2025; Cahill, 2006; Janowsky, 2006; Luine and Frankfurt, 2020)]. An important contributor to AD-related cognitive decline may be the sharp reduction in E2 levels that occurs with aging, most prominently in women, but also in men. However, in aging men, testosterone is still produced and continues to be converted to estradiol via aromatization, leading to higher E2 levels in aged males than in postmenopausal females (Janicki and Schupf, 2010). Low testosterone levels have also been associated with increased phosphorylated tau protein levels in both men and women (Sundermann et al., 2020). Perplexingly, these changing E2 levels in aged men have been reported to increase, have no effect on, and decrease AD risk (Geerlings et al., 2003; Kusters et al., 2023; Li et al., 2014). Studies have also reported that higher levels of endogenous E2 did not affect AD risk in both men and women (Geerlings et al., 2003). In women, undergoing surgical or natural menopause before the age of 45 increased the risk of mild cognitive impairment and dementia (Coughlan et al., 2023; Hogervorst, 2013), suggesting that reductions in circulating E2 may be detrimental, with menopause-associated increases in estrone and follicle-stimulating hormone levels potentially contributing to AD risk (Xiong et al., 2022).

Despite the widespread use of hormonal contraceptives (HCs), few studies have examined their influence on AD pathology. HCs suppress endogenous ovarian hormones while introducing synthetic hormones, which can lead to different outcomes for AD risk. Some studies described a link between HC use and reduced AD risk (Li et al., 2016), whereas others reported no effects (Najar et al., 2020). These opposing reports may be related to differences in HC formulation, age of onset/offset, and duration of use. Together, these findings reveal that the direction of sex hormone effects on AD risk is varied, highlighting a need for additional research to determine the specific effects of endogenous, exogenous, and synthetic sex hormones on neurobiological mechanisms underlying AD.

Much of the current literature focuses on sex hormone influences on the cortex, amygdala, and especially the hippocampus in AD; however, recent studies have shed light on the role of the brainstem in AD pathophysiology (Ji et al., 2021; Lee et al., 2015). Sex/gender differences are reported in hippocampal connectivity to the brainstem, which appears significantly stronger in MCI males than in MCI females (Williamson et al., 2022), but the effects of sex hormones on these brainstem circuits remain largely unexplored. Given the conflicting results across sexes, there is a need for sex-stratified biomarker research, extending beyond the traditionally studied circuits and mechanisms to include brainstem networks, to clarify how hormone dynamics contribute to AD.

## 3 The NI RLN3/RXFP3 signaling pathways: implications for AD

### 3.1 NI is activated by neurogenic stress and affected by AD-related neuropathology

Preclinical studies suggest that NI-based circuits contribute to arousal and locomotion (Lu et al., 2020; Ma et al., 2017b), anxiety-like behaviors (Olucha-Bordonau et al., 2018; Ryan et al., 2011), learning and memory (Ma et al., 2009; Nategh et al., 2015; Ryan et al., 2011; Szõnyi et al., 2019; Zichó et al., 2023), and sleep (Smith et al., 2011), and NI neurons are activated by neurogenic stressors in rodents (Calvez et al., 2016b,c; Kumar et al., 2017; Rajkumar et al., 2016; Ryan et al., 2011; Smith et al., 2011; Walker and Lawrence, 2017). The NI contains a heterogeneous population of mainly GABAergic and fewer glutamatergic neurons, with ascending forebrain projections (Ma et al., 2007; Nardone et al., 2024). A large proportion of NI neurons are peptidergic in nature in rats and mice, containing RLN3 (Ma et al., 2007; Nasirova et al., 2020; Smith et al., 2010; Tanaka et al., 2005), neuromedin-B (Lu et al., 2020; Nasirova et al., 2020), and cholecystokinin (Gugula et al., 2025; Olucha-Bordonau et al., 2003; Szlaga et al., 2022) in overlapping and separate populations, respectively (Gugula et al., 2025; Lu et al., 2020). RLN3 expression in the rat NI is regulated by neurogenic stressors and the stress peptide, corticotropin-releasing hormone (CRH) (Banerjee et al., 2010; Ma et al., 2013), and RLN3-positive neurons express corticotropin-releasing hormone receptor type 1 (CRHR1) (de Ávila et al., 2024; Ma et al., 2013; Tanaka et al., 2005). Recently, the anatomy of the human NI was characterized using RLN3 as a neurochemical marker, and these studies also revealed CRHR1 mRNA expression, and an accumulation of tau pathology colocalized with RLN3 immunoreactivity in NI neurons of AD patients (de Ávila et al., 2024).

In rat and mouse brain, RLN3 acts via the inhibitory G_*i*/*o*_-protein-coupled receptor, RXFP3, (Liu et al., 2003; Ma and Gundlach, 2007) which is highly expressed by somatostatin (SOM) interneurons in the hippocampus (Haidar et al., 2017, 2019; Rytova et al., 2019) and cortex. Postmortem tissue from patients with AD pathologies revealed that SOM interneurons are among the earliest cell types lost in AD (Gabitto et al., 2024). Furthermore, mice with RXFP3 deleted in the hippocampal dentate gyrus demonstrate spatial memory impairments (Haidar et al., 2017), supporting the idea that the NI modulates cognitive functions through its peptidergic signaling pathways.

Currently, functional studies have employed synthetic RXFP3-selective RLN3 peptide analogues, almost all examined only in rodent models [see (Riches et al., 2025)]. In rats, central administration of the agonist R3(A11–24, C15A) reduced anxiety- and depressive-like behaviors (Ryan et al., 2013), while RXFP3 stimulation increased hippocampal theta activity and improved memory performance, and these effects were blocked by the antagonist R3(BΔ23–27)R/I5 (Olucha-Bordonau et al., 2018; Vafaei et al., 2024). These findings indicate that RXFP3 signaling affects behavioral and physiological functions disrupted in AD. However, sex-related differences in RLN3 expression and RXFP3 responsiveness (de Ávila et al., 2021), add complexity, as pharmacological manipulation of this system does not produce equivalent outcomes in males and females (Calvez et al., 2016a; Lenglos et al., 2015). Although the preclinical data are encouraging, features of current peptide-based compounds, such as poor brain access when delivered systemically, restrict their translational use. Similarly, small molecules enter the brain more readily and offer a more practical approach for targeting central RXFP3, but candidates with suitable affinity, selectivity and pharmacodynamics have not been identified so far (Gay et al., 2022; Guan et al., 2025). However, advances in structural analysis, ligand engineering, and medicinal chemistry are expected to develop novel RXFP3-directed compounds (Luo et al., 2025; Riches et al., 2025) appropriate for studies of AD-related mechanisms.

### 3.2 Relationship between RLN3 levels, estrous cycle, menopause, and AD?

RLN3 mRNA levels in NI neurons fluctuate across the estrus cycle in female rats, with the lowest levels during the proestrus phase when E2 reaches its highest levels in circulation (de Ávila et al., 2021). In addition, NI neurons of female rats express the estrogen receptors ERα, ERβ, and G-protein-coupled receptor 1 (GPER1); and among the three receptors, *Gper1* mRNA expression was the most abundant (de Ávila et al., 2021). In brain slices from adult female rats, 33% of “type 1” NI neurons [i.e., express A-type potassium current (Szlaga et al., 2022)] were inhibited by E2, including RLN3-positive neurons (de Ávila et al., 2021). Further studies are needed to understand if the decrease in E2 levels during menopause could alter RLN3 levels and NI neuron activity in the human NI, and whether human NI neurons express estrogen receptors.

Notably, multimodality brain imaging, including magnetic resonance imaging, has revealed sex/gender differences in AD development, suggesting that the preclinical AD phase is earlier in the female aging process than in males and coincides with the endocrine transition of perimenopause (Mosconi et al., 2018). Additionally, plasma RLN3 levels in female and male AD patients are significantly lower than in non-demented control subjects of the same sex/gender (Dammer et al., 2022), further suggesting a link between RLN3 signaling and AD.

## 4 Sex/gender differences in AD pathology and therapeutics

### 4.1 Sex-specific AD biomarkers and therapeutics

Although AD is often viewed as an age-related disease, its greater prevalence in women than men remains unresolved. Sex-specific interventions for AD are essential because the clinical and neuropathological presentations differ substantially between men and women. Interventions that ignore hormonal context may be less effective in women, especially during menopause, when rapid declines in estrogen and progesterone increase vulnerability. Hormone replacement therapy (HRT) is a common treatment for women in menopause and comes in different formulations, e.g., various estrogens, estrogens alone, or combined estrogens and progestins. HRT is often considered to be beneficial for women with MCI or AD due to the reputation of E2 as a neuroprotective factor; however, research over the last two decades illustrates that its effects may depend on various factors including but not limited to dose, type, route of administration, cyclicity, age, and whether the individual is an APOE ε4 allele carrier (Gravelsins and Galea, 2025). It has been suggested that early initiation around menopause may confer neuroprotection, whereas later treatment at age 65 or older may be ineffective or even worse, harmful, e.g., leading to increased and more rapid accumulation of tau (Ali et al., 2023; Coughlan et al., 2025). However, low-dose, transdermal E2 may reduce dementia risk in women 65 or older (Baik et al., 2024), demonstrating that the beneficial effects of HRT may rely on the route of administration, as transdermal and vaginal applications appear more effective than oral treatment. HRT formulation is also critical, as there are reported negative effects with combined E2 and progestin HRT in older women (Shumaker et al., 2003). In addition, HRT has been shown to specifically benefit AD patients carrying the APOE ε4 allele, improving memory and increasing amygdala and hippocampal volumes (Saleh et al., 2023). Together, these findings highlight the importance of identifying AD biomarkers that account for sex/gender, age, and hormonal status that could lead to more individualized and effective HRT and related strategies.

A possible example of a sex-specific biomarker is the RLN3/RXFP3 signaling system. In rodent studies, RLN3 mRNA levels fluctuate across the estrous cycle (de Ávila et al., 2021), and RXFP3 modulation is associated with stress responses, arousal, and cognitive flexibility. Although human translational data are limited, this system’s sensitivity to hormonal state makes it an excellent candidate for further research. If similar hormonal fluctuations occur across the menstrual cycle or during perimenopause, RLN3/RXFP3 signaling could contribute to differential vulnerability or resilience in women, and clinical research is warranted to evaluate this hypothesis. Confirming that NI RLN3/RXFP3 signaling is a sex hormone-sensitive system in humans and understanding how it is altered by not only endogenous, but also exogenous or synthetic hormones may identify mechanisms linking this pathway to AD pathology. Targeting RLN3/RXFP3 pathways may therefore offer a therapeutic approach tailored to hormonal status. Further research is needed to explore the RLN3/RXFP3 system in aging women and across reproductive transitions, and to determine whether modulation of this pathway can slow AD risk or progression.

### 4.2 Sex/gender differences in the NI RLN3/RXFP3 system in AD - future directions

Despite strong evidence of the contribution of the NI RLN3/RXFP3 system to learning, memory and related processes in rodents (Gil-Miravet et al., 2021; Haidar et al., 2017, 2019; Ma et al., 2009; Nategh et al., 2015; Szõnyi et al., 2019; Zichó et al., 2023), the impact of AD on NI function and its RLN3/RXFP3 signaling system in validated animal models of AD has not been investigated. Therefore, it is timely to promote such preclinical studies to better understand the impact of AD pathology on this system and the contribution of NI RLN3/RXFP3 signaling to memory decline or preservation, and associated sex/gender differences. For example, research incorporating sex-stratified spatial transcriptomics and targeted manipulation of NI network signaling and circuit-level analyses (Lu et al., 2020; Ma et al., 2017a; Szõnyi et al., 2019) will be key to understanding how this pathway functions in male and female AD/non-AD brains.

Furthermore, clinical studies using fMRI with a suitable NI marker, and the detection of RLN3 levels in patient cerebrospinal fluid (and plasma) and post-mortem brain regions are warranted [see e.g., (Duron et al., 2018; Oztan et al., 2018; Srivastava and O’Reilly, 2025)]. Correlating neuropsychiatric symptoms with peripheral and central levels of NI markers, including RLN3, in AD and control subjects should promote a better understanding of the nature of any related cognitive decline and co-morbid behavioral changes, such as anxiety and depression (Rabl et al., 2025). Finally, these future studies should assess the influence of sex and other hormone changes, which will be crucial when developing therapies that best account for sex differences in AD susceptibility and disease progression. Together, these basic and translational studies should reveal whether the NI RLN3/RXFP3 system can guide the development of sex-informed experimental and therapeutic approaches aimed at modifying AD-related disease processes and symptoms.

## Footnotes

1 In this article, sex is defined as biological attributes such as chromosomes and anatomy that distinguish an individual as a male, female, or intersex, whereas gender refers to the sociocultural construct that include roles, behaviors, and identities of males, females, and gender diverse individuals (Canadian Institutes of Health Research, 2023). Due to the interconnectedness of these terms that render their influences inseparable, both with be combined and referred to as “sex/gender.”
